# Statistical Simulation of the Switching Mechanism in ZnO-Based RRAM Devices

**DOI:** 10.3390/ma15031205

**Published:** 2022-02-05

**Authors:** Usman Isyaku Bature, Illani Mohd Nawi, Mohd Haris Md Khir, Furqan Zahoor, Abdullah Saleh Algamili, Saeed S. Ba Hashwan, Mohd Azman Zakariya

**Affiliations:** 1 Department of Electrical and Electronic Engineering, Universiti Teknologi PETRONAS, Bandar Seri Iskandar 32610, Perak, Malaysia; illani.nawi@utp.edu.my (I.M.N.); harisk@utp.edu.my (M.H.M.K.); furqan_18000022@utp.edu.my (F.Z.); abdullah_17008405@utp.edu.my (A.S.A.); saeed_17007858@utp.edu.my (S.S.B.H.); mazman_zakariya@utp.edu.my (M.A.Z.); 2 Department of Computer and Communications Engineering, Abubakar Tafawa Balewa University Bauchi (ATBU), Bauchi 0248, Nigeria

**Keywords:** numerical modeling, COMSOL simulation, thermodynamic process, ZnO RRAM, operation voltage

## Abstract

Resistive random access memory (RRAM) has two distinct processes, the SET and RESET processes, that control the formation and dissolution of conductive filament, respectively. The laws of thermodynamics state that these processes correspond to the lowest possible level of free energy. In an RRAM device, a high operating voltage causes device degradation, such as bends, cracks, or bubble-like patterns. In this work, we developed a statistical simulation of the switching mechanism in a ZnO-based RRAM. The model used field-driven ion migration and temperature effects to design a ZnO-based RRAM dynamic SET and RESET resistance transition process. We observed that heat transport within the conducting filament generated a great deal of heat energy due to the carrier transport of the constituent dielectric material. The model was implemented using the built-in COMSOL Multiphysics software to address heat transfer, electrostatic, and yield RRAM energy. The heat energy increased with the increase in the operating power. Hence, the reliability of a device with high power consumption cannot be assured. We obtained various carrier heat analyses in 2D images and concluded that developing RRAM devices with low operating currents through material and structure optimization is crucial.

## 1. Introduction

**Figure 1:**
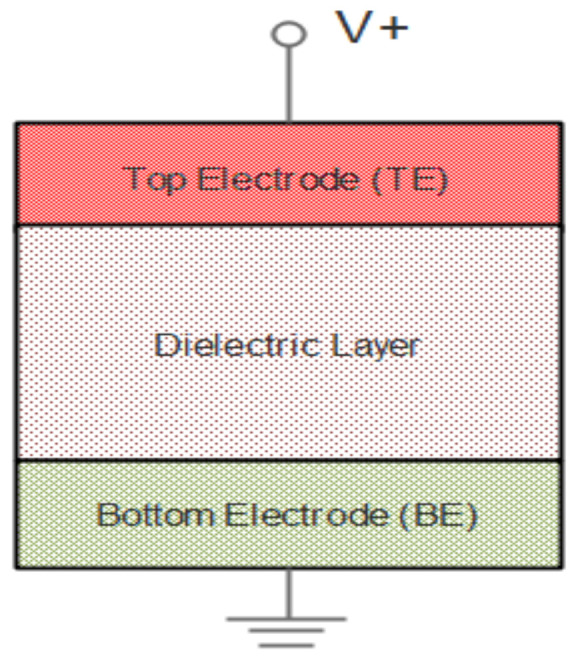
The capacitor-like structure of an RRAM device.

Resistive random access memory (RRAM) based on metal oxide is a promising candidate for the future of nonvolatile memory technology [1]. An RRAM device has a capacitive metal/insulator/metal structure, as shown in Figure 1. Because of their low operating voltage and power, fast switching (10 ns), high density, and excellent scalability, RRAMs are emerging as promising candidates for universal memory [2,3,4]. Various materials have been used to demonstrate resistive switching (RS) behaviors. Among them, ZnO material has been studied extensively and shown to be promising [5,6,7,8,9,10,11]. In RRAM design, ZnO has been established as one of the suitable materials for RRAM devices and has been identified as a multifunctional device that exhibits a significant amount of oxygen vacancy (Vo), which helps in the conductive filament (CF) formation [12,13]. Finding effective methods to monitor the switching efficiency is critical in RRAM design. Understanding resistive switching mechanisms and defining key factors to monitor switching characteristics are crucial to achieving this goal. Several analytical and compact analytical models to explain RS behavior have been developed [14,15,16,17,18]. Understanding transport and switching systems, on the other hand, remains a significant problem. Many researchers have used the formation and rupture of conductive filaments (CFs) in the oxide layer to clarify RS action; however, the switching mechanism is still blurred [19,20]. This work aims to provide memristor users with a simple, comprehensive physical model of switching tools using COMSOL Multiphysics Analytical Software and MATLAB (LiveLink^TM^).

## 2. Thermodynamic Modelling Process

The nano-sized metal films utilized in RRAM devices are fundamental because they produce a short thermalization time [21]. Therefore, the system reaches thermal equilibrium more quickly than the RS time. Thus, in this work, we applied the development of the thermodynamic phase to describe the RS properties. In the evolution of the thermodynamics phase, the stages are constantly evolving when the device is exposed or under an external applied electric field. Thus, during the development period, the system minimizes its energy. Niraula and Karpov considered the thermodynamic theory of RRAM through three concepts [19]: insulating (I), unstable conducting (UC), and metastable conducting phases (MC). These stages illustrate the three (3) categories of RRAM’s operation; insulation, RESET and SET phases.

**Figure 2:**
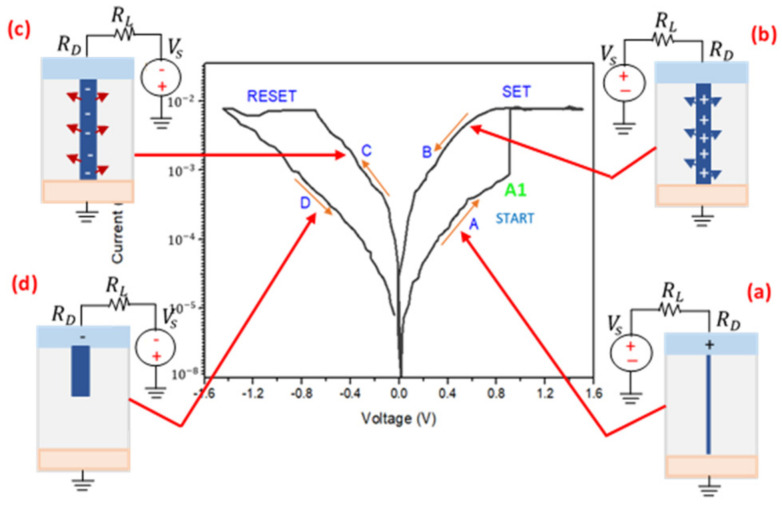
Typical bipolar switching (I-V) curve, similar to the one obtained in the TiN/ZnO/Pt device under sweeping mode [13], and schematic of the processes (**a**–**d**) during the formation and dissociation of CF in RRAM [15]. From the inset: (**a**) shows the formation of cylindrical CF after the applied positive bias as a SET process; (**b**) shows when the device is ON/LRS, and the blue arrows depict the generated radial electric field within the device vicinity; (**c**) shows when the device gained opposite polarity, and the red arrows depict the unfavorable states as a RESET process; and (**d**) shows when the device is transformed to an insulating state (after the dissociation of CF) OFF/HRS. Note: the inset and the pictures are arranged in the SET to RESET processes’ cycle (cycling along (**a**–**d**)).

Furthermore, RRAM toggles between two separate states (SET and RESET), in which both processes are empirically endothermic, as shown in Figure 2. The SET process toggles the device resistance to a low resistance state (LRS) and is known as the ON state, as shown in Figure 2a, whereas the RESET process toggles the device resistance to a high resistance state (HRS) and is known as the OFF state, as shown in Figure 2d. As a result, an intermediate state must exist that is neither ON nor OFF. The insulating state and dielectric medium possess the least energy as compared to ON or OFF states. Hence, this state could be termed as the intermediate state that further assists in distinguishing the other states (HRS and LRS).

We considered the concept of the radial electric field that polarizes the thin film’s environment. First, when a positive bias is swept from 0 to +1.2 V to the 10 nm ZnO dielectric layer, it will create a strong electric field. The dielectric layer will gain a significant amount of electrostatic energy. As such, the layer will become polarized and transform into a conducting layer, since the existing electrical dipoles or/and the charge defects Vo obtain sufficient electrical charge from the externally applied bias polarity. This process is known to be influenced by field induced nucleation (FIN) at the threshold point [22]. The polarity at this point is termed as the threshold voltage (V_T_), as shown at point (A1) in Figure 2. Hence, the electroformed nucleus will longitudinally develop a CF, shunting the device as assumed in Figure 2a. This phenomenon is a sudden process due to the fast transformation of the device’s resistance. The growth of the CF will be subdued when the electrostatic energy drops. The developed CF also charges apart from the conduction of electricity. It produces a strong electric field within its neighborhood, which will polarize the vicinity, as shown in Figure 2b. The current flowing through the CF will cause the system to gain thermal energy due to Joule heating [23]. The CF will radially grow until it reaches a stable radius that equals the system’s minimum free energy (FE) [24]. Thus, increasing the power further will cause the CF’s radius to increase in size.

It is worth noting that, after the radially formed CF has finally shunted the device, then the load resistance (R_L_) will surpass the device resistance (R_D_), denoted as (R_L_>>R_D_). Thus, the current will develop for fixed device voltages, as shown in Figure 2 (point A1 to SET), known as a set voltage (V_SET_). At this point, the corresponding current that allows the total growth of the CF’s radius is termed as the SET current (I_SET_), also known as the compliance current (I_CC_). The polarized vicinity of the charged CF will still be maintained even when the power is disconnected. When the bias polarity is reversed to change the device back to the HRS and OFF state (reversing from 0 to 1.2 V), the charged CF will start to acquire charges opposite to the previously applied polarity. Therefore, the charged CF will oppose the vicinity’s polarization, and the surroundings will gain electrostatic energy, as shown in Figure 2c. The radially formed CF begins to dissociate when the magnitude of the device’s voltage reaches the set voltage value. At this point, the voltage and current are termed the reset voltage (V_RESET_) and reset current (I_RESET_), respectively. The dissolution of the metastable CF compensates for the unfavorably introduced stimulus, resulting in a narrow insulating gap as shown in Figure 2d. Hence, the device’s resistance increases significantly, forcing the device into an insulating phase known as the HRS and OFF state. At this point, the device’s resistance surpasses the load resistance and is denoted as (R_D_>>R_L_). This process is known as gap nucleation in the thermodynamic model. Hence, it is valid to employ the thermodynamic model to enumerate the RS of ZnO-based RRAM devices.

## 3. Numerical Model and Specifications

**Figure 3:**
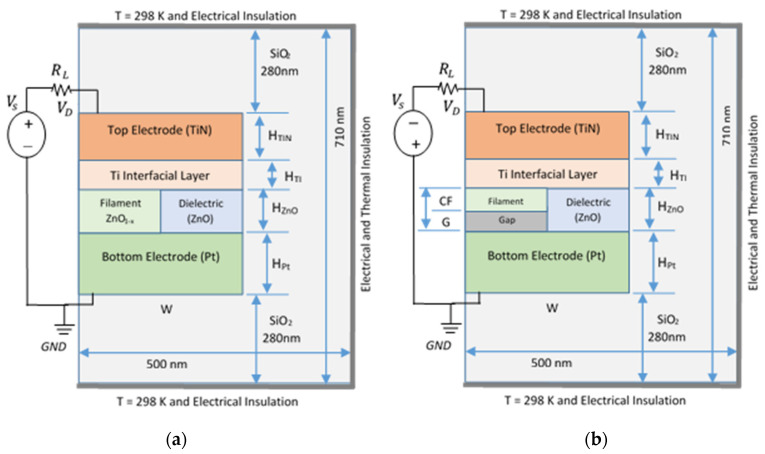
The TiN/Ti/ZnO/Pt device schematic (figures are not sketched to their true scales). (**a**) A diagram of the SET and ON device models. (**b**) A diagram of the OFF and RESET device models. In addition, the sketches depict various boundary conditions, material layers, and symmetrical parameters.

In RRAM’s RS operation, the SET process comprises the CF nucleation process and its longitudinal growth. On the other hand, the RESET process embroils the nucleation and the growth of the gap. However, the nucleation process is stochastic. In this work, we employed COMSOL Multiphysics to investigate the thermodynamic processes. COMSOL Multiphysics was used as a simulation software to compute the temperature and electrical distributions that can be utilized to obtain reliable phase configurations for various source voltages. Hence, the evolution of Vo in the device using this simulation software was described via the electronic conduction and Joule heating during the evolution of the “cone region” that depicted the actual drift and diffusion flux. However, in COMSOL Multiphysics, the physical governing equations are solved using the finite element method. As a result, we solved certain heat electromagnetic partial differential equations to obtain our device’s electrical field and temperature distributions. Various plots of the electrical potential distribution and temperature domain plots were realized and MATLAB (LiveLink^TM^, MathWorks Inc, Natick, MA, USA) was used to establish some of the device’s characteristics. We considered the configuration with a 10nm radius of TiN/Ti/ZnO/Pt multi-layered RRAM structure, as shown in Figure 3, and this shows the reduction of the 3D to 2D axisymmetric geometry that illustrate the rotational symmetry; a similar device pattern was considered by Niraula et al. [15]. The material’s parameters are shown in Table 1.

Similarly, the device’s material dimensions are listed in Table 2. The dimensions were carefully selected to provide the desired voltage output rage. The dimensions were set on a nano meter (nm) scale and the device was hosted by a silicon layer, as shown in Figure 3.

## 4. Implementation in COMSOL Multiphysics

This work focused on the theoretical statistical simulation of switching of a ZnO-based RRAM based on a thermodynamic model implemented in COMSOL Multiphysics analytical software; the software simulates the RS in the device and produces the device’s thermal and electrical properties. Fortunately, COMSOL Multiphysics solves differential time-dependent and stationary equations when processing the four modules (RESET, SET, OFF, and ON).

**Figure 4:**
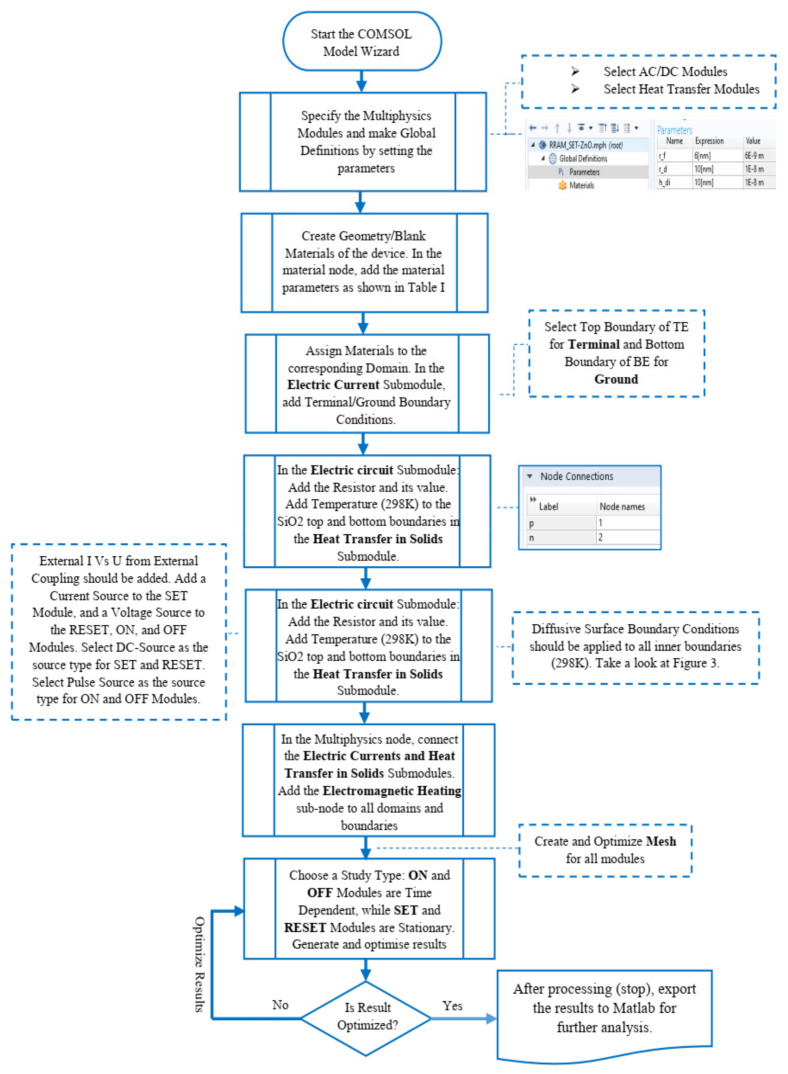
Flow chart of the implementation processes in COMSOL Multiphysics.

The finite element method is intended to solve physical governing equations. As a result, our device’s electrical field and temperature distributions were obtained using COMSOL by solving some heat electromagnetic partial differential equations. The algorithmic steps shown in Figure 4 were used to implement the modules in COMSOL Multiphysics 5.3a. The implementation process starts with opening the model wizard and selecting the space dimension of 2D axisymmetric; all steps are enumerated in Figure 4 to assist in the comprehension of the methods.

COMSOL computes the temperature (*T*) and electric field (*E*) distribution of the device during the electric current, heat transfer in solids, and Multiphysics modules’ execution. Thus, the outputs of these processes can be utilized to determine the device’s free energy (FE). The following sections describe the modules that are considered during the model’s process in COMSOL:

### 4.1. Electric Current Module

During the executions of the four modules, COMSOL solves Ohm’s law, the current conservation law, and the Maxwell law. The expressions in Equations (1) and (2) delineate how the device’s electric field produces a magnetic field within its vicinity.

The stationary expressions shown in Equation (1) are executed during the SET and RESET module processes, while Equation (2) shows the time-dependent version of the expressions solved during the execution of the ON and OFF modules.

(1) $$\nabla .J=0,\;J=\sigma_{c}E,\;E=-\nabla V.$$

(2) $$\nabla .J=0,\;\;J=\sigma_{c}E+\epsilon \frac{\partial E}{\partial t},\;\;E=-\nabla V.$$

where *E* represents the electric field, *J* depicts the electric current density, ∇. shows the divergence operator, ∇ defines the three-dimensional gradient operator, *V* denotes the electric potential, and the electric conductivity is represented by σ_c_.

### 4.2. Heat Transfer in Solids Module

This module computes Fourier’s law, which shows the heat conduction during the SET and RESET executions as represented by the stationary Equation (3); during the ON and OFF module’s executions, the time-dependent Equation (4) is computed, which depicts the distribution of temperature (*T*) on a rigid solid.

(3) $$-\nabla .\left(k\nabla T\right)=Q_{s}$$

(4) $$\rho C_{p}\frac{\partial T}{\partial t}-\nabla .\left(k\nabla T\right)=Q_{s}$$

In the above equations, the absolute temperature in Kelvin is given by *T*, *k* is the thermal conductivity, the specific heat capacity is depicted by $C_{p}$, the electromagnetic heat source is given by $Q_{s}$, and the density is represented by ρ.

### 4.3. Multiphysics Module

In the Multiphysics module, COMSOL computes Equation (5), which represents the system’s electromagnetic heat source (i.e., heat generated by Joule heating). The expression processes couples the electric field and the current density obtained from the electric current and heat transfer in solid modules.

(5) $$Q_{s}=J\cdot E$$

## 5. Simulation Results and Discussion

The simulation results of the ZnO-based RRAM device were obtained from the COMSOL Multiphysics, and the device structure is shown in Figure 3. The device had TiN and Pt as the top electrode (TE) and bottom electrode (BE), respectively. The dielectric material was ZnO, which was deposited on the Pt as reported in research [13]; a Ti interfacial layer was developed between the TE and ZnO to enhance the Vo storage and boost device performance. Generally, Vo is created when the electric field weakens the bonds in the metal oxides. As such, the material’s electrical resistance is upset by the concentration of the generated Vo [35]. Therefore, to simplify and elaborate the simulation mechanism, vacancies were assumed to be distributed in the region of the CF. However, the region of the CF had two parts—the Vo-dominated and Vo-poor regions—while the outer region of the device was considered as a non-conducting region. Therefore, this work designed and elaborated on a ZnO-based RRAM device’s dynamic switching model via the electronic conduction and joule heating mechanisms.

### 5.1. SET Module

**Figure 5:**
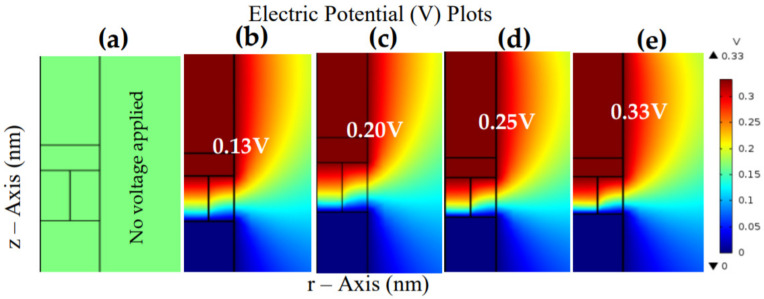
COMSOL images of the SET process of the TiN/Ti/ZnO/Pt device during: (**a**) pristine state, (**b**) threshold voltage, (**c**) the growth of the CF at 0.2 V, (**d**) the growth of the CF at 0.25 V, and (**e**) final evolution of the CF at 0.33 V. These depict the CF growth from the TE to the BE during the externally applied positive polarity sweep.

The SET process is the operation that toggles the RRAM device from a high resistance state to a low resistance state after applying a bias polarity, similarly known as the ON state, that puts the device in a low resistance state due to the growth of the CF. The grown CF will shunt the device’s TE and BE, allowing a free current flow between the electrodes. Thus, the SET process is known as a current-controlled operation. The device shown in Figure 5a (0-V potential) reduced the 3D problem to a 2D equivalent with the vertical coordinate (*z*-axis) and radial coordinate (r-axis); the TiN electrode was connected to an external supply voltage (V_s_). In contrast, the Pt BE was connected to a grounding wire (synonymous to Figure 2). During the fabrication of an RRAM device, the oxide layer is usually exposed to an oxygen-stimulating atmosphere. Thus, it may cause several oxygen defects in the materials. The defects’ neighboring oxygen ion can move to the defective region under the influence of a generated electric field from an external source. Then, the electrical potential energy with Joule heating will be within the vicinity of the moved oxygen ion, and the position can be observed as Vo. As such, the device’s SET and RESET operations can be attributed to the defect migration caused by an electric field and Joule heating.

RRAM is a capacitor-like structure with the dielectric layer as the main switching media. However, RS is either localized as a conductive filament created by dominated soft-breakdown in the dielectric layer, or non-localized as switching across the whole cross-section of the device. From the results obtained in Figure 5, the growth of the “cone shape” seen in Figure 5b–e shows the movement of the Vo defects as a gradual shift and the modification of the conductance in steps on the TE down to BE, as stated by Subhechha et al. [36]. Hence, the defect evolution is accelerated by lowering the potential barrier in the presence of an electric field. Thus, the evolution of the cone tip (red-yellow portion) conformed to this fact and was regarded as the development of the CF [37].

Initially, the device started from a pristine state where no external stimulus was applied, as shown in Figure 5a (no potential applied). Then, a positive sweep voltage was applied from 0 to 0.35 V on the TiN (TE) while grounding the Pt (BE). Figure 5 shows the 2D COMSOL images of the parameterized voltage sweep process considered as a SET process that depicts the growth of the Vo from the TE to the BE. Firstly, during the sweep of the applied source voltage from 0–0.13 V, as shown in Figure 5b, the materials gained significant electrostatic energy (in a sudden process) from the externally applied polarity (Vs). Thus, the layers became polarized and conductive, and this was influenced by FIN at the threshold point known as (V_T_), as shown in Figure 2 (point A1). As such, a longitudinally grown CF was observed to start growing from the TE (red-yellow portion).

**Figure 6:**
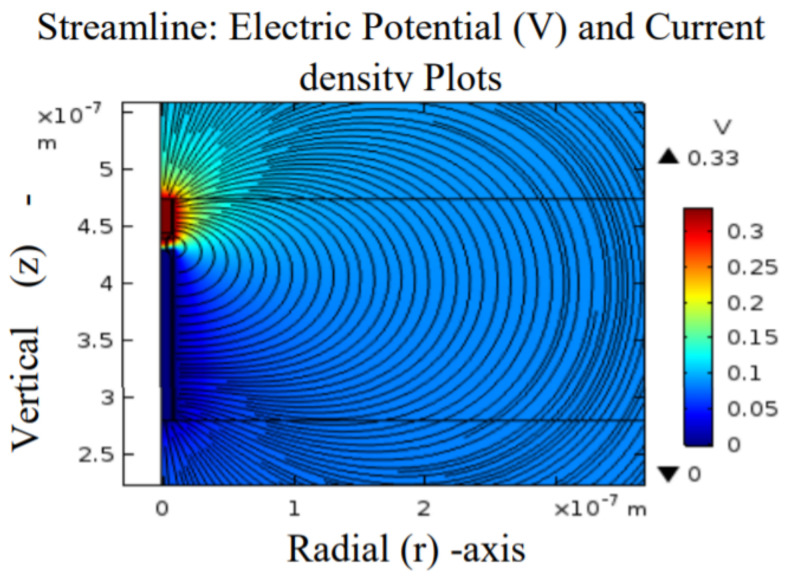
CF’s generated electric field. The generated electric field is due to the electrostatic charges formed by the radially developed CF.

A large number of charge defects (Vo) moved to the BE as the bias was increased, as shown in Figure 5b–e (red-yellow portion), and a conduction cone shape connected the TE and BE at 0.33 V, as shown in Figure 5e (corresponds to Figure 2a). The radially developed CF was charged and generated a strong electric field within its vicinity, as shown in Figure 6 (corresponds to Figure 2b).

**Figure 7:**
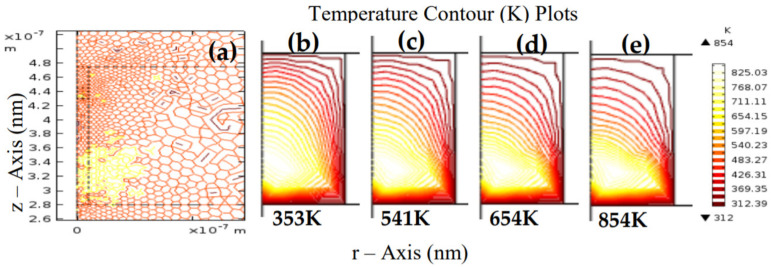
Thermal energy due to Joule heating showing: (**a**) initial ambient temperature, (**b**) the first temperature rise, (**c**) temperature rise during CF growth at 541 K, (**d**) temperature rise at 654 K, and (**e**) temperature rise at 854 K. The device’s temperature rise is due to the current flowing through the radially developed CF; the material gains thermal energy due to Joule heating.

At this point, the current flows through the CF, which makes the device gain thermal energy due to Joule heating. The CF has now grown to a stable state that equates to the system’s FE. The 2D COMSOL images of the device during the Joule heating are shown in Figure 7a–e. The radially developed CF steadily charges and conducts via current flows, and gains the thermal energy that heated its vicinity due to Joule heating.

Hence, the operation voltage of the device has a significant impact on the thermal conductivity of the device. High device operation voltage may cause high thermal conductivity, and consequently material degradation. Therefore, it is prudent to stabilize the device’s operation voltage and guarantee its reliability [38,39]. The dielectric layer of an RRAM device plays a vital role in the device’s performance. It hosts the Vo and electrostatic charges during the device operation. Thus, it is pertinent to analyze the device through the dielectric media.

**Figure 8:**
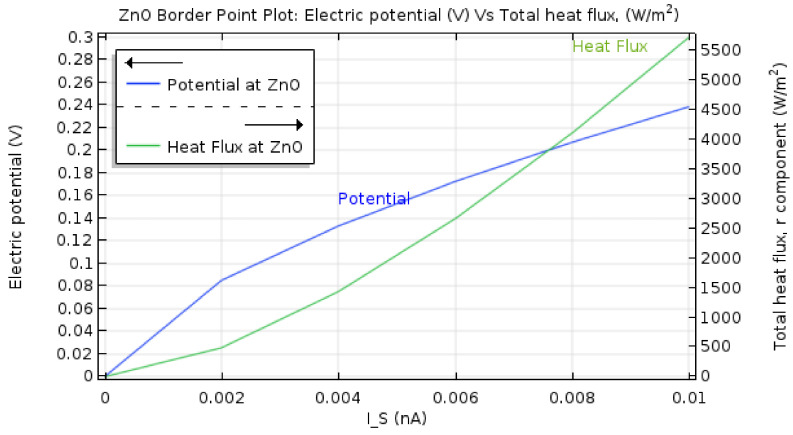
Dielectric layer (ZnO) electrical potential and heat flux characteristics.

Figure 8 shows the dielectric layer’s (ZnO) electrical potential (V) and heat flux (W/m^2^) during the SET process. The inset depicts how the device’s electrical potential and heat flux exponentially increase with the device operating current. Thus, the device’s operational current and voltage must be scaled down to avoid device breakdown or degradation.

### 5.2. RESET Module

The RESET process is the operation that pushes the device into a high resistance state. It is known as the OFF state due to the dissociation of the radially formed CF during the SET state. The RESET process depends on the change in the device’s voltage due to the rupture of the CF; thus, it is a voltage-controlled process.

Based on the previous available research, the model used was a MIM arrangement, as shown in Figure 1, and was created using the COMSOL 2D axisymmetric modules. However, to simplify the analysis, the conductive filament was assumed to be evolving down from the TE to BE during the application of the parameterized scanning voltage, thus producing changes in the temperature, vacancies, electric field, and electric potential. Similarly, the device started from the RESET process model and it was also assumed that the conductive filament was fully conducting at the start of this process.

**Figure 9:**
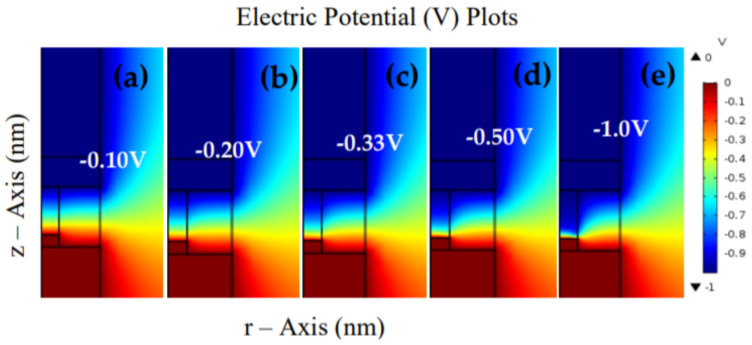
2D COMSOL images of the RESET process of TiN/Ti/ZnO/Pt device during: (**a**) initial conditions, (**b**) −0.20 V sweep, (**c**) the reverse voltage at −0.33 V, (**d**) the voltage sweep at −0.50 V, and (**e**) the residual CF at −1.0 V. These images depict the CF rupture during the sweep of the externally applied negative polarity (blue-red portions show the gradual rupture of the CF).

The 2D COMSOL images of the RESET process are shown in Figure 9. Firstly, after the device has been SET operation and in an LRS, the charged CF-polarized environment will maintain its condition even after power disconnection. When the polarity of the bias is reversed from 0 to −1.0 V, then the charged CF will acquire charges that are opposite to the initial polarity. Thus, it will oppose the environment’s polarization and gain electrostatic energy, as shown in Figure 9a (similar to Figure 2c). The migration of ions and Vo usually depends on the thermal and electrical fields. Further application of the bias to the top region of the CF may lead to energetic movement and intense vibration of the atomic lattice around the CF region. Thus, the increase in the temperature due to Joule heating is enough to make the Vo move towards the TE and transform the morphology of the CF (known as CF rupture); the whole process is shown in Figure 9a–e.

**Figure 10:**
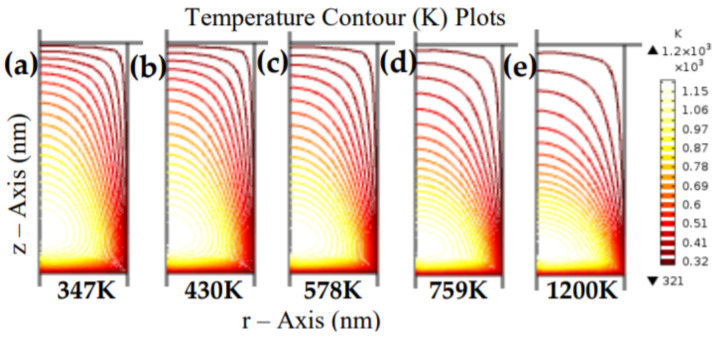
Reset thermal energy due to Joule heating displaying: (**a**) initial reset ambient temperature after the RESET process, (**b**–**e**) the RESET temperature rise due to the application of reverse polarity (the charged CF opposes the vicinity’s polarization). The device’s temperature rise is due to charged CF acquiring charges opposite to the initial polarity; as such, the radially developed CF is dissociated and thus creates gaps.

Furthermore, the RESET process is not an abrupt process, and it is much slower than the SET process because the diffusion is quite contrary to the drift movement. A great deal of thermal heat is generated along the CF region due to the strenuous movement of the Vo and ions to overcome the effect of the unfavorably introduced stimulus; the COMSOL thermal heat images generated during the RESET process are shown in Figure 10. Thus, these results further indicate that the device’s operation voltage level plays a significant role in controlling the RRAM’s internal temperature.

### 5.3. Effect of Thermal Transport

Moreover, we can deduce from Figure 7, Figure 8, and Figure 10 that the insulating layer gains less thermal conductivity compared to that shown in the filament zone. From Figure 3, the filament length can be assumed to be *h*, as shown to be embedded in the dielectric layer (ZnO) in our case, and a source voltage is applied on the TE. The dielectric layer is sandwiched between the TE and BE, as shown in Figure 1. Consider the expression shown in Equation (3) in the heat transfer in solids module; this Fourier’s law shows the heat conduction during the SET and RESET executions. By combining Equations (3) and (5) that represent the system’s heat generated by Joule heating, Equations (6) and (7) that are known as the steady-state equation with Joule heating are yielded [16].

(6) $$-\nabla \left(k\nabla T\right)=Q_{s}=J\cdot E$$

Similarly, it can be expressed as:

(7) $$-\nabla \left(k\nabla T\right)=J\cdot E$$

When $\frac{\delta T}{T}\prime \prime \frac{\delta k}{k}$, we consider the thermal conductivity constant as further details are shown in [40]. Furthermore, we employed the Ohm’s law expression shown in Equation (1) to produce the heat conduction equivalence shown in Equations (8) and (9).

(8) $$-\nabla \left(k\nabla T\right)=\left(\;\sigma_{c}E\right)\cdot E$$

(9) $$\nabla^{2}T=-\frac{\sigma_{c}}{k}E^{2}$$

We considered the Wiedemann–Franz–law as the thermal conductivity mechanism [41] and used the Sommerfeld value [42] of the Lorenz number to compute the thermal conductivity of the CF and electrodes via Equation (10):

(10) $$L=\frac{k}{\sigma T}=L_{o}=\frac{\pi^{2}{\left(k_{B}\right)}^{2}}{3e^{2}}=2.44\times 10^{-8}W\Omega K^{2}$$

where *k_B_* denotes the Boltzmann constant, $L_{o}$ is the Sommerfeld value, and *e* is the electron charge.

Therefore, the heat conduction equation in terms of the Lorenz number *L* is given as:

(11) $$\nabla^{2}T=-\frac{\sigma_{c}}{k}E^{2}\;≅\nabla^{2}=-\frac{\sigma_{c}}{kT}E^{2}\equiv \nabla^{2}=-\frac{1}{LT}E^{2}$$

This shows that the heat transport across the 1D filament is proportional to the square of the electric field generated and varies inversely to temperature. The heat conduction in the RRAM device is important for device performance; hence, controlling the thermal heat within the CF neighborhood is crucial. The heat transport process significantly influences the device’s generated electric field and temperature rise. Thus, operation at a low voltage should be the best alternative, since a high power of operation can cause device degradation and failure [39,43].

Furthermore, the heat conduction expression in Equation (11) can be processed further to describe the relationship between the SET temperature and its voltage [16]. Since the RS we considered here through the thermodynamic analysis involves the SET voltage and its temperature, Equation (12) is suitable to describe the relationship between the two quantities [16].

(12) $$V_{SET}\approx \sqrt{\frac{L}{3}}T$$

**Figure 11:**
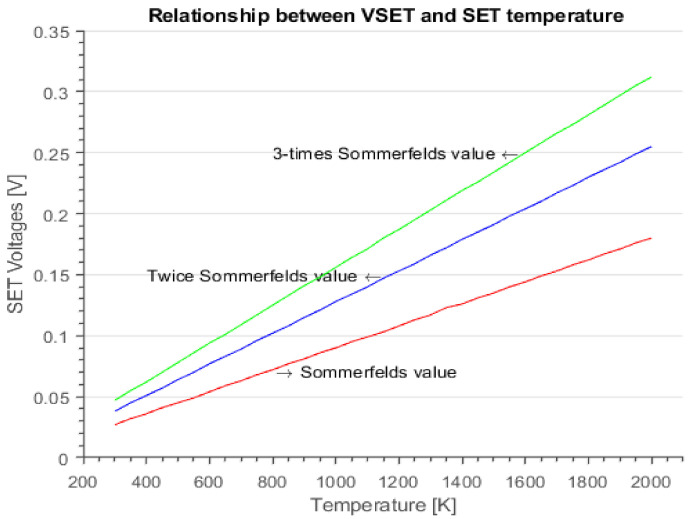
Relation between the SET voltage and temperature.

However, during RRAM operation (SET or RESET), the charge and drift of the oxygen ions and vacancies in the dielectric (oxide) requires an external applied voltage pulse. From Equation (11), using *L* as the Lorentz number, the *V_SET_* at room temperature was 0.027 V, which is close to the ions’ room temperature kinetic energy (*kT*) (0.026 eV). Therefore, since *kT* is the amount of heat needed to increase a system’s thermodynamic entropy, in our device the 0.027V *V*_SET_ would be the minimum amount of voltage pulse required at room temperature to increase the system’s thermodynamic entropy that will charge and drift the oxygen ions and the vacancies in the oxide. Hence, for numerical estimation of the *V_SET_*, we considered Figure 7e having a maximum temperature *T* = 854 K with the effects of the thermal boundary resistance, and two times the value of the Sommerfeld value of the Lorenz number was calculated as $L=4.88\times 10^{-8}W\Omega K^{2}$, for which the solution yields the value of $V_{SET}≅0.11$ V, as low voltages were also reported in some devices [44,45]. Moreover, as shown in Figure 11, one can deduce that the expression in Equation (12) predicts that an RRAM device with a low Sommerfeld value of the Lorenz number could operate with a lower $V_{SET}$ value [16].

**Figure 12:**
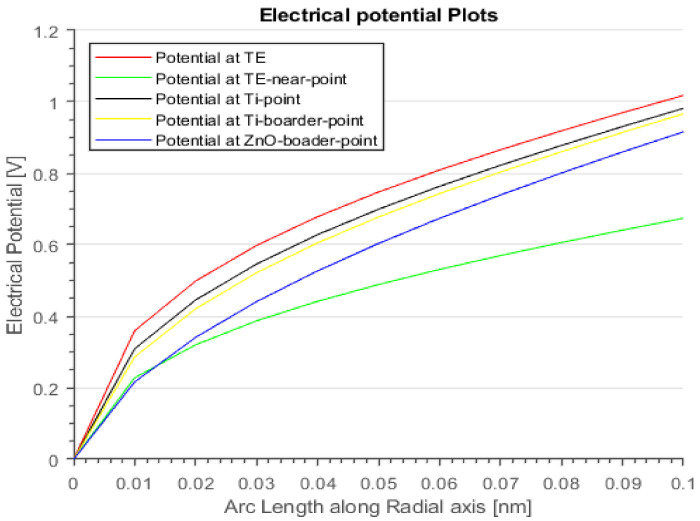
Thermal boundary effect analysis of the materials.

The effect of temperature can be further noticed if boundary mismatch is considered. Two dissimilar materials usually possess different crystal lattice and unit cell structures. The disparity in the lattice at the interface causes transfer resistance and non-uniform temperature distribution across the interface. This non-uniform border temperature and resistance mismatch concept are termed thermal boundary resistance (TBR). TBR at the filament/electrode and filament/insulator junctions causes the filament to heat up to a higher temperature. As shown in Figure 12, the heat conduction to the electrode and insulator regions is obstructed. Then, there will be two boundary temperatures at the junction: one from the filament edge and another on the electrode or insulator side.

Moreover, temperature has an exponential effect on the diffusivity of a material. Since the rate of diffusion increases with the rise in the device energy, this increases the molecular energy and the spontaneous spreading of particles as a statistical issue [46]. The formation of Vo in the ZnO layer may be essential to the creation of oxygen gas at the anode of TiN/Ti/ZnO/Pt. The Kröger–Vink notation can be used to express the electrochemical reaction of Vo and gas creation at the anode:

(13) $$O_{o}^{x}\to V_{o}^{.}+2e^{-}+\frac{1}{2}O_{2}$$

However, interstitial or vacancy diffusion increases exponentially with an increase in temperature, usually denoted as:

(14) $$N_{V}=N_{C}e^{\left(-\frac{E_{V}}{kT}\right)}$$

Equation (14) is a chemical reaction that shows the effect of temperature on the diffusion of the defect via the switching layer. $N_{V}$ is the number of the defect or concentration per cubic centimeter; $E_{V}$ represents the amount of energy required to create a vacancy in cal/mol, J/mol, or eV/atom; k is known as the Boltzmann constant and is usually taken as $8.62\times 10^{-5}{\;\mathrm{eVK}}^{-1}$ or $1.38\times 10^{-23}{\;\mathrm{JK}}^{-1}$; and k is the effective temperature of the CF. $N_{C}$ denotes the number of lattices site per cubic centimeter and can be processed as $\frac{\rho }{A}N_{A}$, where A denotes the atomic mass, ρ is the density, and $N_{A}$ is Avogadro’s constant.

Therefore, for the numerical approximation of the number of defects ($N_{V}$), we considered the various values of the temperatures obtained in Figure 10, $N_{C}=4.81\times 10^{22}{\;\mathrm{cm}}^{-3}$, and the $E_{V}$ stated by Ali et al. [47]. Considering a dielectric temperature of 430 K as shown in Figure 10b, $k\;\sim \;8.62\times 10^{-5}{\;\mathrm{eVK}}^{-1}$, $E_{V}\;\sim \;0.239\;\mathrm{eV}$, and $N_{V}\;≅\;7.62\times 10^{19}\;{\mathrm{cm}}^{-3}$. Similarly, several other values of dielectric temperatures, such as 400 K, 410 K, and 420 K were used to estimate the defect concentration across the material as $4.69\times 10^{19}\;{\mathrm{cm}}^{-3}$, $5.56\times 10^{19}\;{\mathrm{cm}}^{-3}$, and $6.64\times 10^{19}{\;\mathrm{cm}}^{-3}$, respectively. This is consistent with the established facts [16,48] and suggests that the larger the temperature, the larger the diffusion of the vacancies across the ZnO dielectric. Alternatively, the temperature-activated defect diffusivity may occur through the Arrhenius defect migration law [46,48]:

(15) $$\sigma \left(n_{D},\;T\right)=\sigma_{o}exp^{\left(-\frac{E_{AC}}{kT}\right)}$$

where $n_{D}$ denotes the concentration of particle is, $\sigma \left(n_{D},\;T\right)$ is the electrical conductivity, the activation energy for conduction is denoted by $E_{AC}$, and the Boltzmann constant is represented by k.

Moreover, with the increase in RRAM integration density, effects such as self-heating may become more prominent and lead to reliability problems. Hence, to address this issue, there is a need to address RRAM thermal distribution as it relates to self-heating; the thermal conduction equation approach has been solved elsewhere [48] as one of the remedies to this problem.

Additionally, the effects of the electric field distribution and operating voltage as the device’s size shrinks have been challenging to the realization of a large scale RRAM memory architecture; these are issues that may lead to cross-talk interference and cause errors during RRAM read operations [49]. As the device’s size shrinks (1 ${\mu m}^{2}\;\mathrm{or}\;\mathrm{below}$) a great deal of reliability and stability issues may arise and hinder the optimum performance of the device. The effect of the electric field due to device’s size reduction may be understood by current density and the effect of the ZnO dielectric constant nature; the thermionic effect of the ZnO layer can be described via the Schottky emission formula [50]:

(16) $$J=A^{**}T^{2}\exp\left[\frac{-q\left(\emptyset_{B}-\sqrt{\left(qV/4\pi \varepsilon_{i}d_{sl}\right)}\right)}{kT}\right]$$

where the device switching layer thickness is given by $d_{sl}$, $\emptyset_{B}$ is the energy barrier, and $A^{**}$ and T are the Richardson constant and the temperature, respectively.

The permittivity is defined as $\varepsilon_{i}=\varepsilon_{o}k$, where k is the dielectric constant of ZnO, which is around 9. From this relation, it can be shown that the thickness of the dielectric layer and its constant play a great role in determining the current density of the device. Thus, electric field issues caused due to device shrinking are particularly detrimental to the scaling-down of RRAM devices, as they not only consume more power but also cause an excessively high voltage to be applied to neighbouring transistors.

Interestingly, the use of relatively high-k materials to surround the RRAM’s switching layer is capable of stabilizing the electric field distribution and renders the CF as more stable [50]. The surrounding high-k material should have a higher dielectric constant than the device’s switching layer material. Hence, the switching layers of Al_2_O_3_, SiO_2_, and ZnO should have a surrounding material with a higher dielectric constant value, such as HfO_2_ or TiO_2_, as this may keep the forming voltage constant and stabilize the operating power as the device’s size shrinks.

## 6. Conclusions

The evaluation of the fundamentals of resistive switching in ZnO-based RRAM based on the thermodynamic model was proposed. The model was used to study this dynamic process during the resistance transition using field-driven ion migration and the temperature effect. Various COMSOL 2D images showed that the Vo migration in a ZnO-based RRAM device is stimulated by the electric field and Joule heating effect. The current that flows through the CF significantly impacts the device’s thermal energy; hence, a high device operating voltage will increase power consumption and cause device degradation. Therefore, the device’s operation voltage plays a significant role in its reliability. This thermodynamic simulation model may be potentially effective for gaining fundamental perspectives of the microscopic morphological features of the RRAM operation.

## Figures and Tables

**Table 1:** Material parameters used in the model [25,26,27,28,29,30,31,32,33,34].

Material	K [WK^−1^m^−1^]	C_p_ [JKg^−1^K^−1^]	σ [Sm^−1^]	$\epsilon_{r}$	ρ [Kgm^−3^]
TiN	11.9	545	10^6^	−∞ ^1^	5.22 × 10 ^3^
Ti	21.9	522.6	2.5 × 10^6^	−∞ ^1^	4.506 × 10 ^3^
ZnO	49	40.30	7.26 × 10^−7 3^	2.4	5.606 × 10 ^3^
Zn	116	389	1 × 10^7^	4	7.140 × 10 ^3^
ZnO_1−x_	70 ^2^	100 ^2^	2 × 10^−4 2^	3 ^2^	6.500 × 10^3 2^
Pt	77.8	133	9.43 × 10^6^	10	21.425 × 10 ^3^
SiO_2_	1.38	703	10^−14^	3.9	2.2 × 10 ^3^

^1^ 10^6^ was used for practical purpose as an alternative to −∞ [15]. ^2^ Assume a value that lies between ZnO and zinc. ^3^ Value assumed to change after the forming process.

**Table 2:** Dimensions of the device.

Device Measurements	Device I (nm) (ON/SET)	Device II (nm) (OFF/RESET)
H_TiN_	30	30
H_Ti_	5	5
H_ZnO_	10	10
H_ZnO(1−x)_	10	-
H_Pt_	150	150
W	10	10
CF	5	5
G	-	2

## Data Availability

The data reported in this research are available from the corresponding author upon request.
